# Hippo/YAP signaling pathway protects against neomycin-induced hair cell damage in the mouse cochlea

**DOI:** 10.1007/s00018-021-04029-9

**Published:** 2022-01-19

**Authors:** Maohua Wang, Ying Dong, Song Gao, Zhenhua Zhong, Cheng Cheng, Ruiying Qiang, Yuhua Zhang, Xinyi Shi, Xiaoyun Qian, Xia Gao, Bing Guan, Chenjie Yu, Youjun Yu, Renjie Chai

**Affiliations:** 1 Department of Otolaryngology, Head and Neck Surgery, The First People’s Hospital of Foshan, Affiliated Foshan Hospital of Sun Yat-Sen University, Hearing and Balance Medical Engineering Technology Center of Guangdong, Foshan, 528000 China; 2 State Key Laboratory of Bioelectronics, Department of Otolaryngology Head and Neck Surgery, Zhongda Hospital, School of Life Sciences and Technology, Jiangsu Province High-Tech Key Laboratory for Bio-Medical Research, Southeast University, Nanjing, 210096 China; 3 Department of Otolaryngology, Head and Neck Surgery, Affiliated Drum Tower Hospital of Nanjing University Medical School, Jiangsu Provincial Key Medical Discipline (Laboratory), Nanjing, 210008 China; 4 Department of Otolaryngology, Head and Neck Surgery, The Affiliated Hospital of Yangzhou University, Yangzhou University, Yangzhou, 225000 China; 5 Department of Otolaryngology, Head and Neck Surgery, Affiliated Jiangning Hospital of Nanjing Medical University, Nanjing, 211100 China; 6 Department of Otolaryngology, Head and Neck Surgery, Clinical Medical College, Yangzhou University, Yangzhou, 225001 China; 7 Department of Otolaryngology Head and Neck Surgery, Sichuan Provincial People’s Hospital, University of Electronic Science and Technology of China, Chengdu, 610072 China; 8 Co-Innovation Center of Neuroregeneration, Nantong University, Nantong, 226001 China; 9 Institute for Stem Cell and Regeneration, Chinese Academy of Science, Beijing, China; 10 Beijing Key Laboratory of Neural Regeneration and Repair, Capital Medical University, Beijing, 100069 China

**Keywords:** Aminoglycosides, Hair cells, Apoptosis, Protection, Hippo/YAP signaling pathway, Sensorineural hearing loss (SNHL)

## Abstract

The Hippo/Yes-associated protein (YAP) signaling pathway has been shown to be able to maintain organ size and homeostasis by regulating cell proliferation, differentiation, and apoptosis. The abuse of aminoglycosides is one of the main causes of sensorineural hearing loss (SSNHL). However, the role of the Hippo/YAP signaling pathway in cochlear hair cell (HC) damage protection in the auditory field is still unclear. In this study, we used the YAP agonist XMU-MP-1 (XMU) and the inhibitor Verteporfin (VP) to regulate the Hippo/YAP signaling pathway in vitro. We showed that YAP overexpression reduced neomycin-induced HC loss, while downregulated YAP expression increased HC vulnerability after neomycin exposure in vitro. We next found that activation of YAP expression inhibited C-Abl-mediated cell apoptosis, which led to reduced HC loss. Many previous studies have reported that the level of reactive oxygen species (ROS) is significantly increased in cochlear HCs after neomycin exposure. In our study, we also found that YAP overexpression significantly decreased ROS accumulation, while downregulation of YAP expression increased ROS accumulation. In summary, our results demonstrate that the Hippo/YAP signaling pathway plays an important role in reducing HC injury and maintaining auditory function after aminoglycoside exposure. YAP overexpression could protect against neomycin-induced HC loss by inhibiting C-Abl-mediated cell apoptosis and decreasing ROS accumulation, suggesting that YAP could be a novel therapeutic target for aminoglycosides-induced sensorineural hearing loss in the clinic.

## Introduction

Hearing loss is one of the most common sensory defects in humans. According to the World Health Organization (WHO) report in 2018, approximately 466 million people suffer from hearing loss worldwide [1]. There are three main types of hearing loss—sensorineural hearing loss, conductive hearing loss, and mixed hearing loss—of which sensorineural hearing loss accounts for the vast majority of cases [2]. While noise exposure, aging, long-term use of ototoxic drugs [3], and viral infection can lead to varying degrees of sensorineural hearing loss, irreversible damage to cochlear hair cells (HCs) is the fundamental cause of sensorineural hearing loss [4, 5]. More than 150 drugs have ototoxic effects [6, 7], and aminoglycoside antibiotics are one of the most common ototoxic drugs causing sensorineural hearing loss in the clinic [8]. HCs sense the stimulation of sound waves, and mammalian HCs lack regenerative capacity; thus, once HCs are damaged, permanent hearing loss is inevitable.

The Hippo signaling pathway was first discovered in the study of the Drosophila Warts (WTS) gene mutation in 1995 [9]. Later studies found that mutations of Salvador (SAV) [10], Hippo (HPO) [11], and Mob as tumor suppressor (MATS) [12] could also cause excessive growth of tissues and organs. Given their close relationship, these genes are collectively referred to as the Hippo signaling pathway [13]. In 2005, Yorkie (YKI), a major effector factor, was found to play a regulatory role in the Hippo signaling pathway, and conditional knockout of YKI was found to inhibit tissue overgrowth caused by mutations of upstream effectors such as WTS, SAV, and HPO [14]. Similarly, the Hippo signaling pathway is highly conserved in mammals, and its core effector factors include Mammalian ste20-like protein kinase 1/2 (MST1/2) (same as HPO), large tumor suppressor kinase 1/2 (LATS1/2) (same as WTS), and Yes-associated protein (YAP) (same as YKI) [15]. As a core effector of the Hippo signaling pathway in mammals, conditional knockout of YAP can also inhibit cell overgrowth [16]. As the transcriptional coactivator, YAP lacks a domain that can directly bind to DNA, so it needs to be combined with downstream transcription factors to enter the nucleus and carry out its biological functions [17]. Tea domain transcription factor (TEAD), a major transcription factor, interacts with YAP and plays a regulatory role [18].

The Hippo/YAP signaling pathway has been shown to be able to maintain organ size and homeostasis of the internal environment by regulating cell proliferation, differentiation, and apoptosis, and it is known to play an important role in the occurrence and development of cancer [15, 19–23]. There are three regulatory mechanisms of YAP that are regulated by Hippo signaling, namely phosphorylation [24], protein–protein interactions [25], and the competitive binding of VGLL4 to the TEAD transcription factor [26, 27]. When the Hippo signaling pathway is on, activated MST1/2 phosphorylates LATS1/2, leading to the phosphorylation of YAP, which then either stays in the cytoplasm or degrades [13] and participates in cell apoptosis and differentiation [16]. In contrast, when the Hippo signaling pathway is off, YAP enters the nucleus after dephosphorylation and forms a complex with TEAD to participate in cell proliferation [28]. In addition to numerous upstream factors regulating the Hippo signaling pathway, the cascade itself has a negative feedback regulation system. YAP can increase the expression of NF2, LAST2, and MST1 by binding to TEAD in the nucleus, which has a negative feedback regulatory role on YAP [13].

Recently, an increasing number of scholars have found that YAP activation plays an important role in organ regeneration and regenerative medicine [29]. Indeed, YAP activation can promote the proliferation of retinal precursor cells and the differentiation of pigment cells. In contrast, conditional knockout of YAP could reduce cell proliferation, promote cell apoptosis, and induce pigment deposition leading to retinal degeneration in mice [30], indicating that the Hippo/YAP signaling pathway plays a critical regulatory role in the development of the retina. Other studies have also found that YAP knockout not only inhibits bile duct proliferation but also enhances hepatocyte necrosis and inhibits hepatocyte proliferation, while YAP activation prevents cholestasis injury in mice [31]. The loss of YAP in the nervous system further aggravates lysophosphatidic acid (LPA)-induced hemorrhagic hydrocephalus, which is partially recovered after enhanced YAP expression [32]. Upregulation of YAP expression has also been found to promote the healing of skin wounds [33]. Moreover, enhanced YAP activity can protect the myocardium in acute stress injury [34]. In kidney disease, podocyte injury leads to podocyte death and loss, which results in progressive kidney damage and ultimately kidney failure, and the loss of YAP in podocytes further increases Adriamycin-induced podocyte apoptosis [35]. All of these studies have demonstrated the regulatory role of the Hippo/YAP signaling pathway in tissue protection and regeneration after injury. However, the role of the Hippo/YAP signaling pathway in protecting HCs against damage in the mouse cochlea remains unclear.

In this study, we used the YAP agonist XMU-MP-1 (XMU) and the YAP inhibitor Verteporfin (VP) to regulate the expression level of YAP and investigated the role of the Hippo/YAP signaling pathway in protecting against aminoglycoside-induced cochlear HC damage in vitro. We found that the Hippo/YAP signaling pathway could regulate C-Abl-mediated HC apoptosis and the accumulation of ROS, which protects against neomycin-induced HC loss after neomycin injury. Thus, aminoglycoside-induced cochlear HC damage could be prevented by regulating the Hippo/YAP signaling pathway. This will allow hearing function to be preserved and provide a novel therapeutic target for aminoglycoside-induced sensorineural hearing loss.

## Materials and methods

### Experimental animals

In this study, we used wild-type (WT) FVB mice from the Jackson Laboratory. The mice were raised and provided by the Key Laboratory of “Development and Disease-related Genes,” Ministry of Education, Southeast University. FVB mice are inbred WT mice that have the following characteristics: uniform genetic background, good consistency of experimental results, strong reproductive capacity, large litter size, and low spontaneous tumor rate. Therefore, inbred mice are preferred as experimental animals to study gene function or disease mechanisms. All animal procedures were performed according to the protocols approved by the Animal Care and Use Committee of Southeast University, and were consistent with the National Institutes of Health Guide for the Care and Use of Laboratory Animals. All efforts were made to reduce the number of animals used and to minimize their suffering.

### Tissue culture

Cochleae were dissected from postnatal day (P)3 mice and cultured as previously reported [36]. The cochlear sensory epithelium was isolated and seeded intact on a glass coverslip coated with Cell-Tak (Corning, 354240) and cultured in DMEM/F12 (Gibco, 11330-032) supplemented with 2% B27 (Invitrogen, 17504-044), 1% N-2 (Invitrogen, 17502-048), and 50 mg/ml ampicillin (Sigma-Aldrich, P0781) [37]. In the experimental group, the cochlear tissues were pretreated with 2 μM VP (dissolved in DMSO, Sigma-Aldrich, SML0534-5ML) or 1 μM XMU (dissolved in DMSO, Astatech, 43245) for 12 h. Next, 0.5 mM neomycin (Sigma-Aldrich, N6386-5G) was added for 12 h to induce HC damage. After neomycin was removed, the tissues were allowed to recover in serum-free medium for a further 12 h. In the control group, the tissues were recovered in serum-free medium for 36 h without neomycin, XMU, or VP, and the medium was changed every 12 h.

### Immunofluorescence

The TUNEL BrightRed Apoptosis Detection Kit (Vazyme, A111-03), Anti-Cleaved Caspase-3 antibody (Cell Signaling Technology, 9664S), Mito-SOX Red (Life Technologies, M36008), Anti-YAP antibody (Cell Signaling Technology, 12395S, 1:400 dilution), Anti-C-Abl antibody (Affinity Biosciences, AF6038, 1:400 dilution), Anti-Myosin7a antibody (Proteus Biosciences, 25–6790, 1:1000 dilution), Anti-Sox2 antibody (RD, AF-2018, 1:400 dilution), Alexa Fluor 488 donkey Anti-Rabbit IgG (Invitrogen, A-21206, 1:400 dilution), Alexa Fluor 555 donkey Anti-Mouse IgG (Invitrogen, A-31570, 1:400 dilution), Alexa Fluor 647 donkey Anti-Goat IgG (Invitrogen, A-21447, 1:400 dilution), and DAPI (Solarbio, C0060, 1:1000 dilution) were used to measure apoptotic cells and to stain HCs and nuclei. The dissected cochlear tissues were fixed in 4% paraformaldehyde (Sigma-Aldrich, 158127) for 1 h, then washed three times with PBST (1 × PBS [Sigma, P5493] with 0.1% Triton X-100 (Solarbio, 1109F0521). After blocking for 1 h in medium (10% donkey serum, 0.1% Triton X-100, and 1% BSA in PBS at pH 7.2) at room temperature, the samples were incubated with primary antibody diluted in PBT-1 (0.1% Triton X-100, 5% donkey serum, 1% BSA in PBS at pH 7.2) overnight at 4 °C. The samples were washed three times with PBST and then incubated with the secondary antibody diluted in PBT-2 (1% BSA and 0.1% Triton X-100 in PBS at pH 7.2) for 1 h at room temperature. After incubating with secondary antibody, the samples were washed three times with PBST and mounted on glass slides with DAKO (DAKO, S3023). Finally, the fluorescence images were obtained by a confocal microscope (LSM710, Zeiss, Heidelberg).

### Western blot

The dissected cochlear tissues were lysed with RIPA Lysis Buffer (Protein Biotechnology, PP109) plus Phosphatase Inhibitor Cocktails (Roche, 04693132001). The fully reacted tissue mixture was centrifuged at 13,000 rpm for 5–10 min at 4 °C. The supernatant solution was mixed with 5 × sodium dodecyl sulfate buffer (Beyotime, P0015L) at a ratio of 1:4 to obtain a protein sample, and the samples were boiled in water for 15 min. Equal amounts of protein samples were loaded onto a 10% Tris–glycine SDS-PAGE gel, electrophoresed at 120 V for 2 h, and then transferred to a 0.2-μm polyvinylidene difluoride membrane (Millipore, Immobilon ISEQ00010). Following transfer, the membranes were blocked with 5% BSA (Biosharp, 4240), then washed three times with 1 × PBST (1 × PBS [Sigma, P5493] with 0.1% Triton X-100 [Solarbio, 1109F0521]). Protein concentrations were measured using a BCA Protein Quantification Kit (Protein Biotechnology, PP202), with GAPDH as the reference protein. Anti-YAP mouse polyclonal antibody (Cell Signaling Technology, 12395S, 1:400 dilution), Anti-C-Abl rabbit polyclonal antibody (Affinity Biosciences, AF6038, 1:400 dilution), and GAPDH mouse monoclonal antibody (Abcam, ab8245, 1:1000 dilution) were used as primary antibodies. Horseradish peroxidase-conjugated goat anti-rabbit (or anti-mouse) IgG (Abcam, ab6789, ab6721) was used as the secondary antibody. The protein signals were detected using a SuperSignal West Dura chemiluminescent substrate kit (Thermo Fisher Scientific, 34075) on a FluorChem M system (ProteinSimple, San Jose, CA, USA).

### Quantitative real-time PCR (qPCR)

Total RNA was extracted from 8 to 10 whole cochleae using TRIzol Reagent (Protein Biotechnology, PR910). The integrity of the RNA samples was evaluated by the A260/A280 ratio, and the samples were reverse transcribed to cDNA using the Revert Aid First Strand cDNA Synthesis kit (Thermo Fisher Scientific, K1622). The qPCR was performed on an Applied Biosystems CFX96 real-time PCR system (Bio-Rad, Hercules, CA, USA) using the FastStart Universal SYBR Green (Rox) qRT–PCR Master Mix (Roche Life Science, 4913850001). The qRT–PCR conditions were set as follows: 15 s denaturation at 95 °C followed by 40 cycles of denaturation at 95 °C for 15 s, annealing at 60 °C for 60 s, and extension at 72 °C for 20 s. β-actin was used as the housekeeping gene for control. The concentration and purity of RNA and cDNA were determined with a Nano-Drop (Thermo Fisher, 2000). The primer sequences are shown in Table 1.

**Table 1:** The primer sequences were used in this study

Gene	Forward sequence (5′–3′)	Reverse sequence (5′–3′)
*YAP*	ACCCTCGTTTTGCCATGAAC	TGTGCTGGGATTGATATTCCGTA
*C-Abl*	AGCCGCTTCAACACTCTGG	ACACCGTAGATAGTGGGCTTG
*Bax*	TGAAGACAGGGGCCTTTTTG	AATTCGCCGGAGACACTCG
*Caspase8*	ATGGCGGAACTGTGTGACTCG	GTCACCGTGGGATAGGATACAGCA
*Apaf1*	AGTGGCAAGGACACAGATGG	GGCTTCCGCAGCTAACACA
*FADD*	GCTCCAGAATGGGCGAAGTAA	ACGGATGTGCGGAGGTAAAAA
*Bcl-2*	GTCGCTACCGTCGTGACTTC	CAGACATGCACCTACCCAGC
*TEAD-2*	GAAGACGAGAACGCGAAAGC	GATGAGCTGTGCCGAAGACA
*Lats1*	AAAGCCAGAAGGGTACAGACA	CCTCAGGGATTCTCGGATCTC
*P73*	GCACCTACTTTGACCTCCCC	GCACTGCTGAGCAAATTGAAC
*β-actin*	ACGGCCAGGTCATCACTATTG	AGGGGCCGGACTCATCGTA
*Nqo1*	AGGATGGGAGGTACTCGAATC	AGGCGTCCTTCCTTATATGCTA
*Gsr*	TGCACTTCCCGGTAGGAAAC	GATCGCAACTGGGGTGAGAA
*Glrx*	AGTCTGGAAAGGTGGTCGTG	CCATTAGCATGGCTGGACGA
*Sod1*	GGAGCAAGGTCGCTTACAGA	AGTGACAGCGTCCAAGCAAT
*Alox15*	GGCTCCAACAACGAGGTCTAC	AGGTATTCTGACACATCCACCTT

### Cell counts

In the neomycin-treated groups, we counted the number of Myosin7a + HCs that remained under a 40 × microscope. The same procedure was used to quantify cleaved caspase-3 + /Myosin7a + cells, TUNEL + /Myosin7a + cells, and MitoSOX Red + /Myosin7a + cells. In all experiments, only one cochlea from each mouse was used for immunofluorescence and quantification; therefore, n represents the number of mice examined.

### Statistical analysis

Data are shown as the mean ± the standard error of the mean (S.E.M). Statistical analyses were conducted using Microsoft Excel and GraphPad Prism 7 software. The counting data and qPCR data were statistically analyzed by GraphPad Prism 7, while ImageJ was used to count the number of cells in the immunofluorescence map, where the image size, layer, and contrast could be modified. Two-tailed, unpaired Student’s t tests were used to determine statistical significance when comparing two groups, and one-way ANOVA followed by a Dunnett multiple comparison test was used when comparing more than two groups. *p* values < 0.05 were considered statistically significant. The experimental data and images were recombined and typeset using Adobe Illustrator software. Each experiment was repeated at least three times to ensure the accuracy and reliability of the experimental results (*n* ≥ 3). The number of replicates is indicated in each figure legend.

## Results

### Expression and localization of YAP in mouse cochlear HCs

**Fig. 1:**
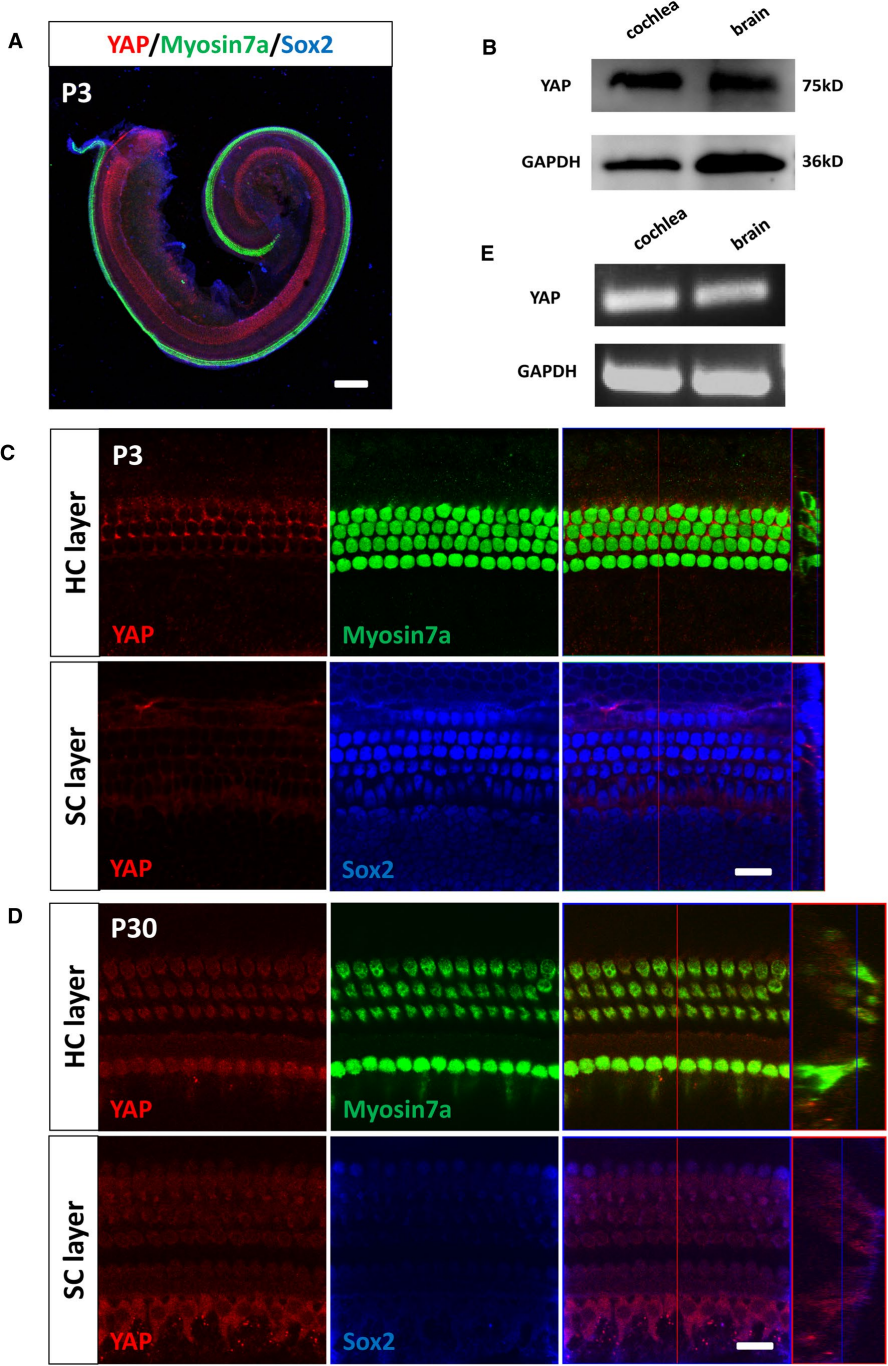
The expression and localization of YAP in mouse cochlear HCs. **A** The cochleae were dissected from P3 WT mice, and immunolabeled with Myosin7a (green), Sox2 (blue), and YAP (red). Immunofluorescence staining showed that the expression and localization of YAP in the P3 WT mouse cochlea under a 5 × microscope. **B** Western blot results showed that YAP was strongly expressed in the mouse cochlea and brain. **C** Immunofluorescence staining showed the expression and localization of YAP in the P3 WT mouse cochlea under a 63 × microscopy. **D** Immunofluorescence staining showed the expression and localization of YAP in the P30 WT mouse cochlea under a 63 × microscope. **C**, **D** Immunofluorescence staining showed that YAP was expressed in the cytoplasm of cochlear HCs. **E** RT-PCR results showed that YAP was strongly expressed in the mouse cochlea and brain. *HC* hair cell, *SC* supporting cell; Scale bar: **A**: 200 μm; **C**, **D**: 10 μm. *N* = 5

To determine the role of the Hippo/YAP signaling pathway in cochlear HC injury protection, we first studied the expression and localization of YAP (a core effector of Hippo signaling pathway) in the mouse cochlea. We dissected the cochleae from postnatal day (P)3 and P30 WT mice and immunolabeled them with the HC marker Myosin7a, the supporting cell (SC) marker Sox2, and DAPI to label the DNA. We first observed the cochlear basilar membrane under a 5 × microscope (Fig. 1A). Immunofluorescence staining showed that YAP was expressed in the cytoplasm of cochlear HCs (Fig. 1C, D). Western blot and RT-PCR demonstrated that YAP was strongly expressed in the P3 mouse cochlea and brain (Fig. 1B, E). These results suggested that YAP plays an important role in the development of HCs in the mouse cochlea, and may be involved in the regulation of related physiological processes in the inner ear, which provides a basis for further study.

### The Hippo pathway is activated and the expression of YAP is decreased in cochlear HCs after neomycin treatment

**Fig. 2:**
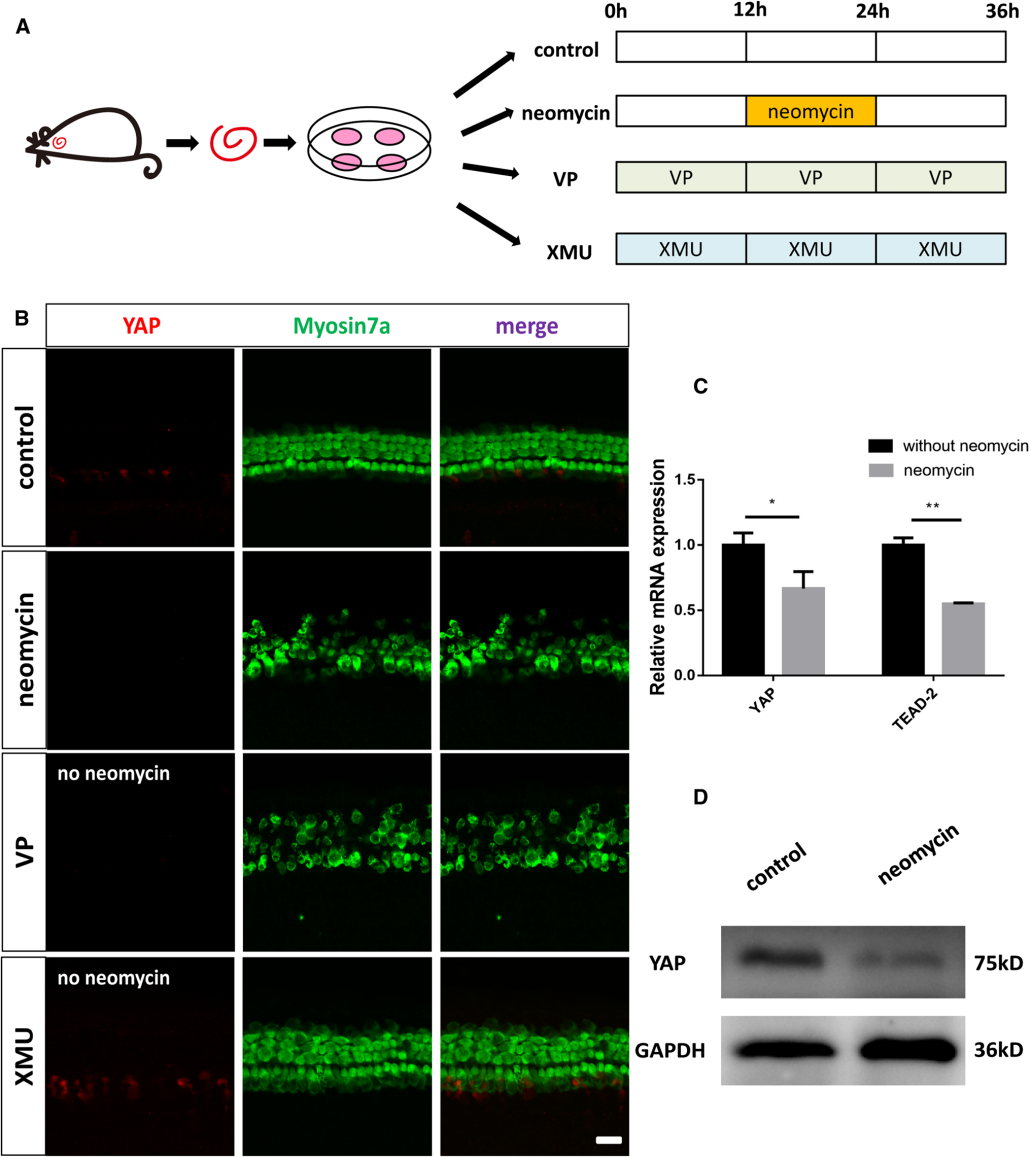
The Hippo pathway is activated and the expression of YAP is decreased in cochlear HCs after neomycin treatment. **A** The dissected cochleae were cultured in vitro with different treatments. In the neomycin-treated group, the cochleae were allowed to recover for the first 12 h, treated with 0.5 mM Neomycin for the next 12 h, and then allowed to recover again for another 12 h. In the VP-treated group, the cochleae were cultured with 5 μM VP for 36 h without neomycin. In the XMU-treated group, the cochleae were cultured with 5 μM XMU for 36 h without neomycin. **B** Immunofluorescence staining showed that the expression level of YAP was decreased in the HCs after neomycin treatment compared with the control group and that the expression of YAP was upregulated in the XMU-treated group and downregulated in the VP-treated group. The HCs were lost after VP treatment. **C** qPCR results revealed that the expression of YAP and the Hippo downstream target gene *TEAD-2* was significantly downregulated after neomycin treatment. **D** Western blot results showed that the expression of YAP was decreased in the HCs after neomycin injury. *p < 0.05, **p < 0.01, ***p < 0.001, *n* = 3. Scale bars = 20 µm

We next explored the expression of YAP and Hippo target genes in the cochlear HCs after neomycin treatment. We dissected the cochleae from P3 WT mice and cultured them with 0.5 mM neomycin for 12 h (Fig. 2A). Both immunofluorescence and western blot results showed that the expression of YAP was decreased in the HCs after neomycin injury (Fig. 2B, D). qPCR results revealed that the expressions of YAP and the Hippo downstream target gene TEAD-2 were significantly downregulated after neomycin treatment (Fig. 2C). These results demonstrated that the Hippo pathway was activated in the cochlear HCs after neomycin injury, indicating that the Hippo/YAP signaling pathway is a protective physiological mechanism against neomycin injury.

### YAP overexpression protects against neomycin-induced HC loss in vitro

**Fig. 3:**
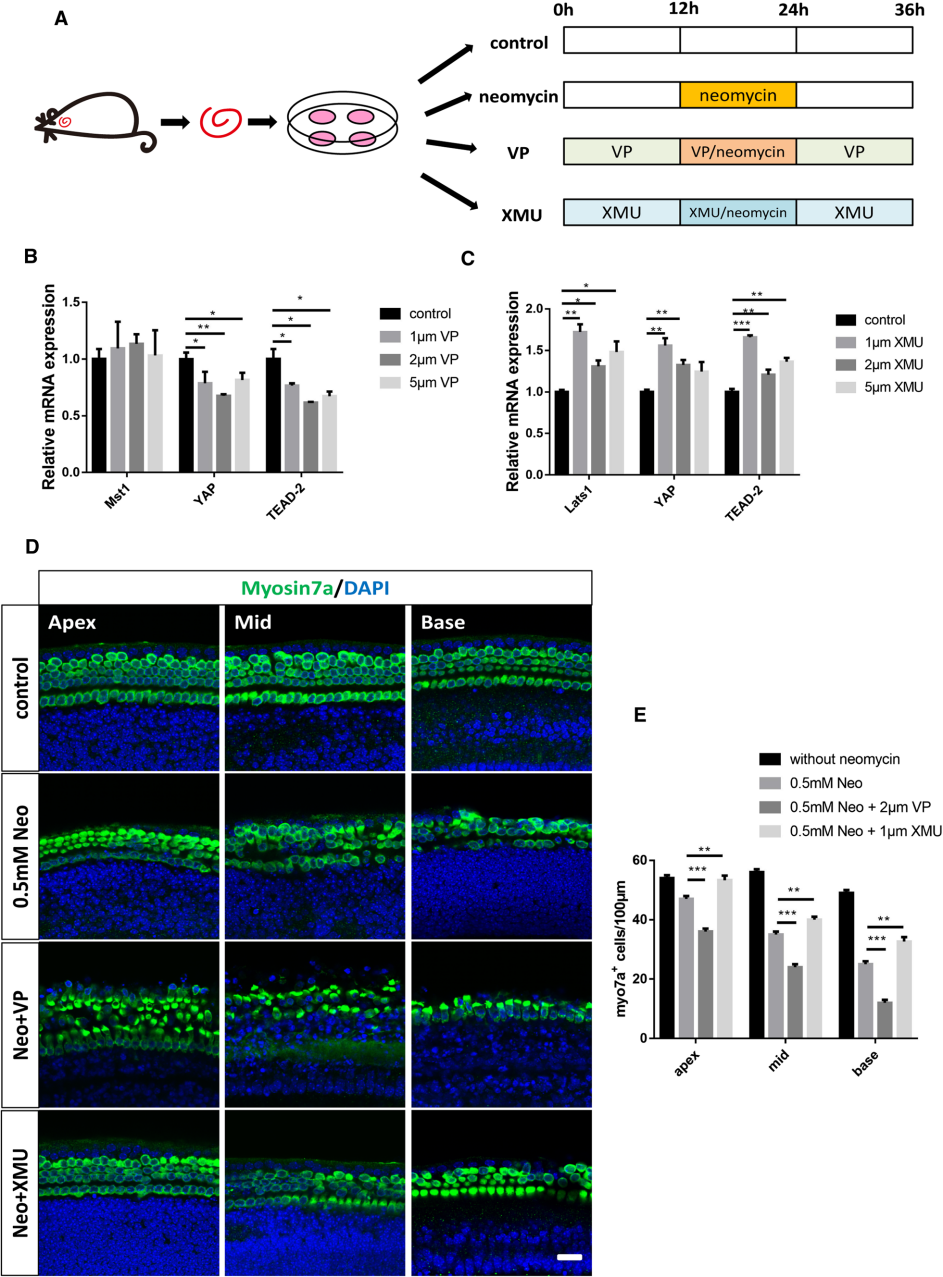
YAP overexpression protects against neomycin-induced HC loss in vitro. **A** Schematic diagram of drug addition in tissue culture (divided into four groups). **B**, **C** The mRNA levels of YAP were analyzed by qPCR after culturing with different concentrations of XMU/VP under the same conditions. The results showed that the optimal concentration of XMU was 1 μM and the optimal concentration of VP was 2 μM. **D** Immunofluorescence staining with Myosin7a and DAPI in the apical, middle, and basal turns of the cochlear basilar membrane after different treatments. **E** Quantification of the numbers of Myosin7a + / DAPI + double-positive cells in **D** showing that the XMU-treated group had significantly reduced HC loss after neomycin treatment. *p < 0.05, **p < 0.01, ***p < 0.001, *n* = 4. Scale bars = 20 µm

To investigate the protective role of the Hippo/YAP signaling pathway against neomycin-induced HC loss, we used the YAP agonist XMU and the YAP inhibitor VP in two independent in vitro experiments. First, cochlear tissues from P3 WT mice were cultured with XMU (5 μM) or VP (5 μM) for 36 h (Fig. 2A). Immunofluorescence staining demonstrated upregulated expression of YAP in the XMU-treated group and downregulated expression of YAP in the VP-treated group. The HCs were lost after VP treatment (Fig. 2B). Both XMU and VP dissolve in DMSO, and it was necessary to find an appropriate drug concentration because excessive concentrations are cytotoxic and will lead to cell apoptosis, which will affect the experimental results. To explore the optimal concentration of XMU and VP, we dissected the cochleae from P3 WT mice and cultured them with different concentrations of XMU and VP for 36 h (Fig. 2A). qPCR results showed that XMU-treated groups had significantly higher expression of YAP and the Hippo downstream target gene TEAD-2 than the control group, while the VP-treated group had significantly lower expression of YAP and TEAD-2 than the control group. Compared to 2 μM and 5 μM, the optimal concentration of XMU was 1 μM, and the optimal concentration of VP was 2 μM compared to 1 μM and 5 μM. Therefore, we chose 1 μM XMU and 2 μM VP pretreatment for 12 h under the treatment conditions in the following experiments (Fig. 3B, C).

We next used 1 μM XMU or 2 μM VP to pretreat the cochleae for 12 h before neomycin exposure. We then treated the cultured cochleae with 0.5 mM neomycin together with 1 μM XMU or 2 μM VP for the next 12 h. Finally, we removed neomycin and treated the cultured cochleae with 1 μM XMU or 2 μM VP for another 12 h (Fig. 3A). The cochleae were immunolabeled with the HC marker Myosin7a and the DNA marker DAPI after culturing the cochleae with different treatments. Immunofluorescence staining showed that the XMU/neomycin-treated group had significantly less HC loss than the neomycin-only group. In contrast, the VP/neomycin-treated group had significantly greater HC loss than the neomycin-only group (Fig. 3D, E). These results demonstrate that YAP overexpression could protect against neomycin-induced HC loss in vitro.

### The Hippo/YAP signaling pathway regulates neomycin-induced HC apoptosis in vitro

**Fig. 4:**
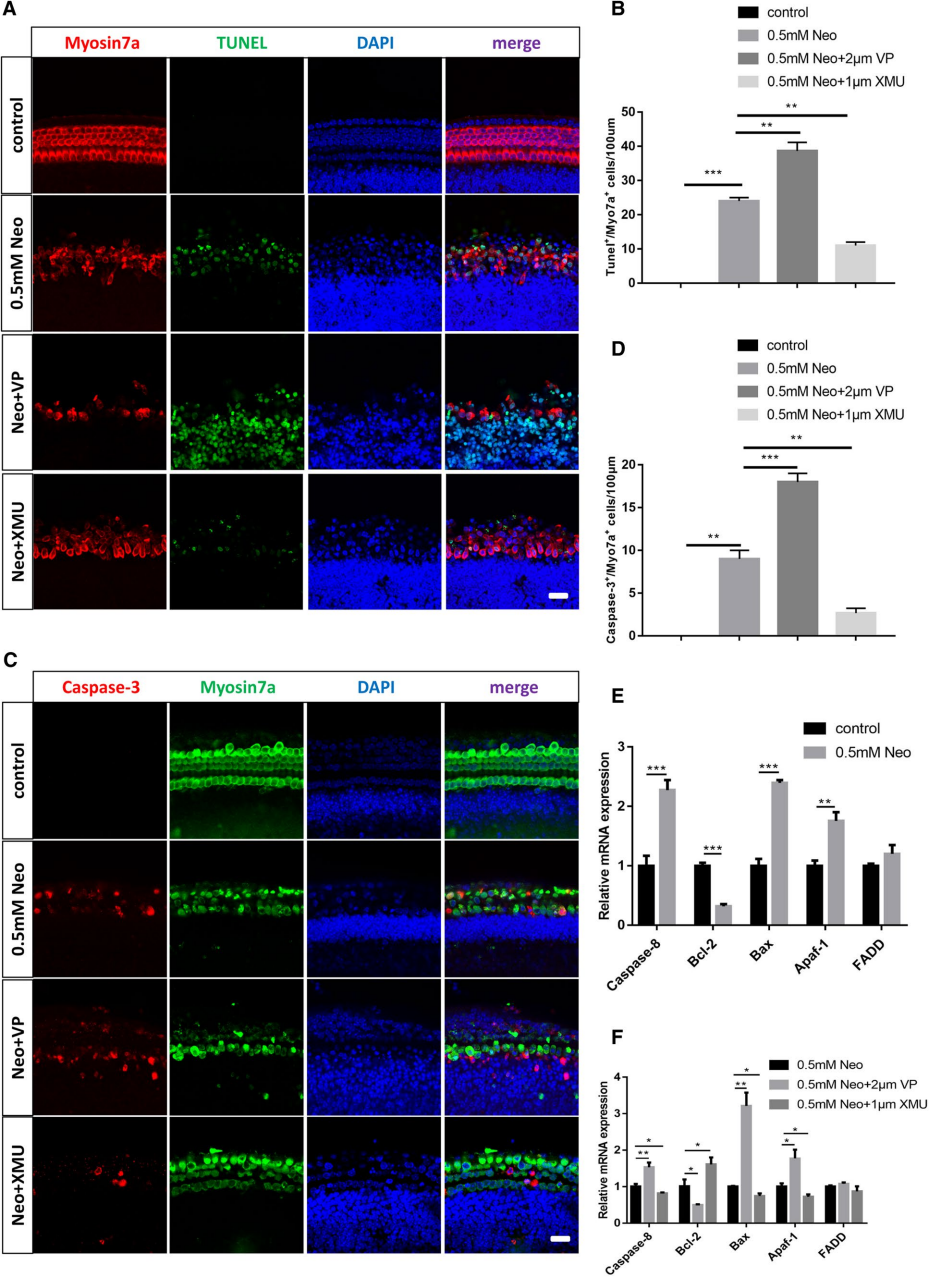
The Hippo/YAP signaling pathway regulates neomycin-induced HC apoptosis in vitro. **A** Immunofluorescence staining with TUNEL and Myosin7a in the middle turn of the cochlear basilar membrane after different treatments. **C** Immunofluorescence staining with cleaved-Caspase3 and Myosin7a in the middle turn of the cochlear basilar membrane after different treatments. **B** and **D** Quantification of the numbers of TUNEL + / Myosin7a + double-positive cells and cleaved-Caspase3 + / Myosin7a + double-positive cells in (**A** and **C**). The numbers of TUNEL + / Myosin7a + double-positive cells and cleaved-Caspase3 + / Myosin7a + double-positive cells were significantly increased in the neomycin-treated group compared with the control group. Moreover, the numbers of apoptotic cells were significantly increased by VP treatment and decreased by XMU treatment. **E** The mRNA levels of apoptosis-related genes were analyzed by qPCR after neomycin treatment. **F** The mRNA levels of apoptosis-related genes were analyzed by qPCR in the VP-pretreatment group and XMU-pretreatment group after neomycin injury. *p < 0.05, **p < 0.01, ***p < 0.001, *n* = 3. Scale bars = 20 µm

We also sought to determine the mechanism by which the Hippo/YAP signaling pathway protects against neomycin-induced HC loss. Previous studies have reported that neomycin kills HCs by inducing apoptosis, in which TUNEL and cleaved-caspase3 were used as markers of aminoglycoside-induced HC apoptosis [38–41]. Therefore, immunofluorescence staining with TUNEL and cleaved-caspase-3 was performed to detect apoptotic cochlear HCs after different treatments. The immunofluorescence results showed that the numbers of cleaved-caspase3 + /Myosin7a + and TUNEL + /Myosin7a + double-positive cells per 100 mm of the cochlea in the middle turn were significantly increased in the neomycin-treated group compared to the control group (Fig. 4A–D). In additon, the numbers of cleaved-caspase3 + /Myosin7a + and TUNEL + /Myosin7a + double-positive cells in the XMU/neomycin-treated group were significantly reduced compared to those in the neomycin-only group. In contrast, when cochleae were pretreated with VP the numbers of cleaved-caspase3 + /Myosin7a + and TUNEL + /Myosin7a + double-positive cells were increased compared to those in the neomycin-only group (Fig. 4A–D).

We next performed qPCR to explore the expression level of apoptosis-related genes in the cochlea after different treatments. The qPCR results demonstrated that the expression of the pro-apoptotic genes *Caspase8*, *Bax* (Bcl-2-associated X protein), and *Apaf1* (Apoptotic Peptidase Activating Factor 1) was significantly increased after neomycin treatment compared to that of the control group, and the expression of the anti-apoptotic gene *Bcl-2* (B Cell Leukemia/Lymphoma 2) was significantly decreased compared to that in the control group (Fig. 4E). Furthermore, the YAP inhibitor VP significantly upregulated the expression of the pro-apoptotic genes *Caspase8, Bax*, and *Apaf1*, and reduced the expression of the anti-apoptotic gene *Bcl-2*. The expression of the pro-apoptotic genes *Caspase8, Bax*, and *Apaf1* was significantly decreased, and the expression of the anti-apoptotic gene *Bcl-2* was increased when the cochleae were treated with the YAP agonist XMU (Fig. 4F). Taken together, the above results suggested that the Hippo/YAP signaling pathway could regulate neomycin-induced HC apoptosis in vitro.

### The Hippo/YAP signaling pathway regulates the ROS levels in cochlear HCs after neomycin injury

**Fig. 5:**
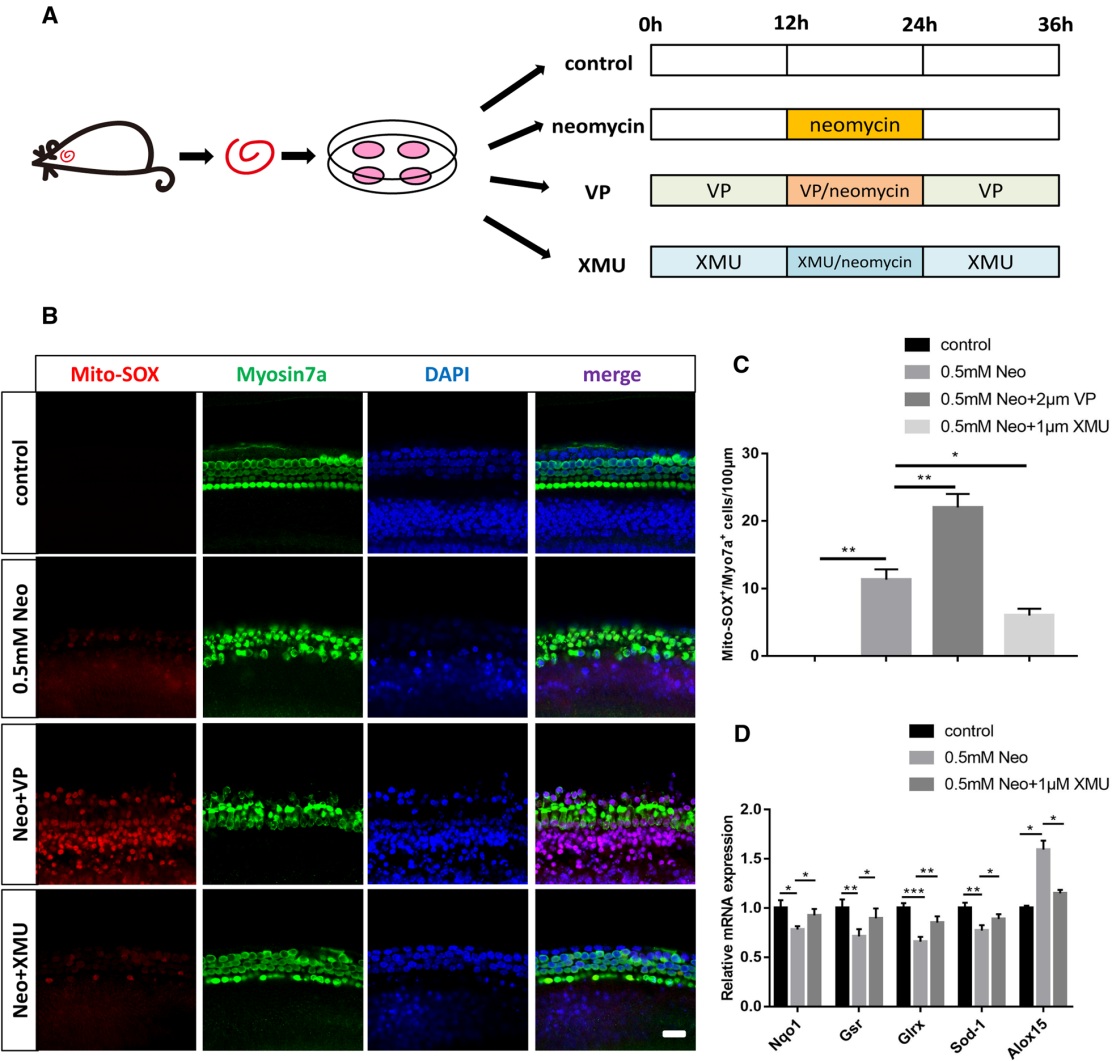
The Hippo/YAP signaling pathway regulates the ROS levels in cochlear HCs after neomycin injury. **A** Schematic diagram of drug addition in tissue culture (divided into four groups). **B** Immunofluorescence staining with Mito-SOX and Myosin7a in the middle turn of the cochlear basilar membrane after different treatments. **C** Quantification of the numbers of Mito-SOX + / Myosin7a + double-positive cells in (**B**). The numbers of Mito-SOX + / Myosin7a + double-positive cells were significantly increased in the neomycin-treated group compared with the control group. In addition, the neomycin-induced oxidative stress was significantly increased by VP treatment and decreased by XMU treatment. **D** The mRNA levels of redox-related genes were analyzed by qPCR in the neomycin-only group and the XMU-pretreatment group after neomycin injury. *p < 0.05, **p < 0.01, ***p < 0.001, *n* = 3. Scale bars = 20 µm

Previous studies have reported that aminoglycoside-induced accumulation of ROS is closely related to HC apoptosis in the mouse cochlea [42–44]. Mito-SOX Red is a redox fluorophore that selectively detects mitochondrial superoxide and can be used to evaluate the ROS levels in different treatment groups [45–48]. To determine the relationship between HC loss and oxidative stress in the mouse cochlea, we dissected and cultured the cochleae from P3 WT mice and then treated them with neomycin together with XMU or VP (Fig. 5A). We used Mito-SOX Red to detect the ROS levels in cochlear HCs by immunofluorescence staining. The immunofluorescence results showed that the number of Mito-SOX + /Myosin7a + double-positive cells per 100 mm of the cochlea in the middle turn was significantly increased in the neomycin-treated group compared to that in the control group (Fig. 5B, C). Additionally, the number of Mito-SOX + /Myosin7a + double-positive cells in the XMU/neomycin-treated group was significantly less than that in the neomycin-treated group. In contrast, when the cochleae were pretreated with VP, the number of Mito-SOX + /Myosin7a + double-positive cells was increased compared to that in the neomycin-treated group (Fig. 5B, C). We next conducted qPCR to investigate the expression level of redox-related genes in the mouse cochlea after different treatments. We found that the expressions of the antioxidant genes *Nqo1* (NAD (P) H: Quinone Oxidoreductase 1), *Gsr* (Glutathione reductase), *Glrs* (Glutaredoxin), and *Sod1* (Superoxide dismutase 1) were significantly decreased after neomycin treatment compared to the control group, and the expression of the pro-oxidant gene *Alox15* (Arachidonate 15-Lipoxygenase) was significantly increased in the neomycin group compared to the control group (Fig. 5D). In addition, the expression of the antioxidant genes *Nqo1*, *Gsr*, *Glrs*, and *Sod1* was significantly increased, and the expression of the pro-oxidant gene *Alox15* was decreased when the cochleae were pretreated with XMU compared to the neomycin group (Fig. 5D). These results demonstrated that the Hippo/YAP signaling pathway regulates the accumulation of ROS in cochlear HCs after neomycin injury.

### C-Abl expression is regulated by the Hippo/YAP signaling pathway in cochlear HCs after neomycin exposure

**Fig. 6:**
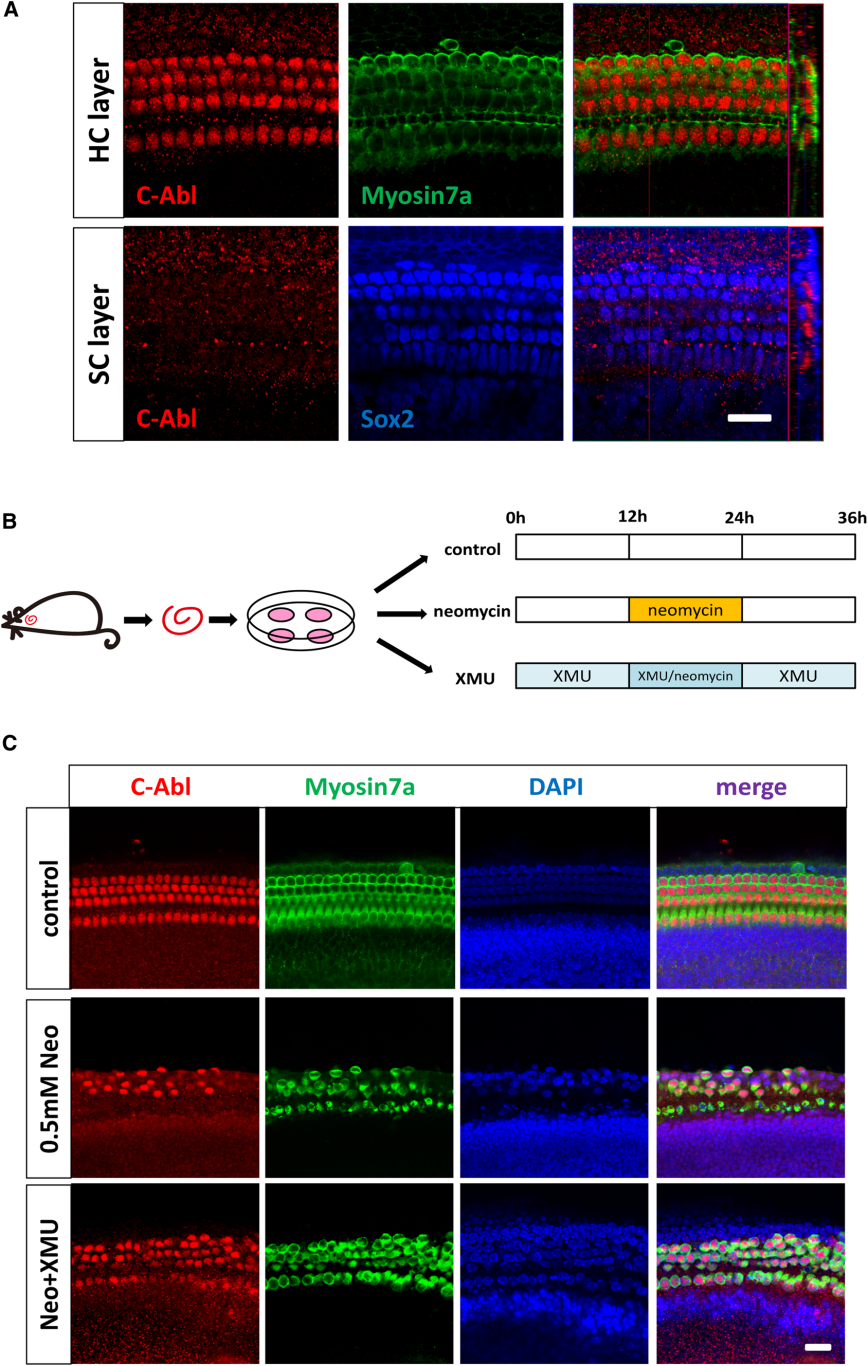
C-Abl expression is regulated by the Hippo/YAP signaling pathway in cochlear HCs after neomycin exposure. **A** The cochleae were dissected from P3 WT mice, and immunolabeled with Myosin7a (green), Sox2 (blue), and C-Abl (red). Immunofluorescence staining showed the expression and localization of C-Abl in the P3 WT mouse cochlea under a 63 × microscope, and C-Abl was expressed in the nuclei of cochlear HCs. **B** Schematic diagram of drug addition in tissue culture (divided into three groups). **C** Immunofluorescence staining with C-Abl and Myosin7a in the middle turn of the cochlear basilar membrane after different treatments. The expression of C-Abl was significantly increased in the neomycin-treated group and decreased in the XMU/neomycin-treated group. *p < 0.05, **p < 0.01, ***p < 0.001, *n* = 3. Scale bars = 20 µm

**Fig. 7:**
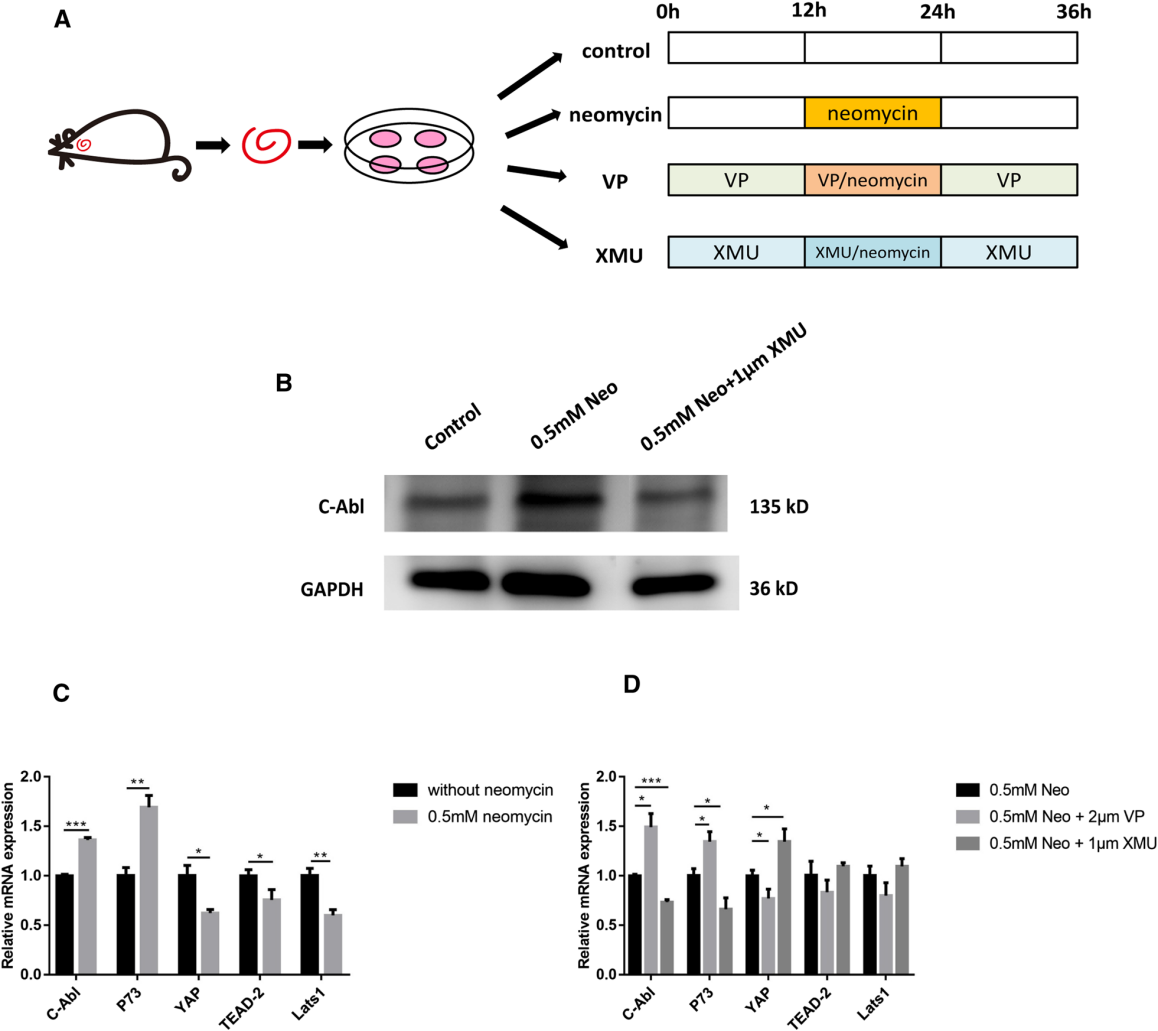
YAP Overexpression inhibits C-Abl-mediated HC apoptosis in cochlear HCs after neomycin damage. **A** Schematic diagram of drug addition in tissue culture (divided into four groups). **B** The cochleae were dissected from P3 WT mice used for Western blot experiment. Western blot showed that the expression of C-Abl was significantly increased in the neomycin-treated group and decreased in the XMU/neomycin-treated group. **C**, **D** The mRNA levels of C-Abl signaling downstream genes were analyzed by qPCR in the different treatment groups. The qPCR results showed that XMU downregulated the expression of C-Abl and p73 and that VP upregulated the expression of C-Abl and p73. *p < 0.05, **p < 0.01, ***p < 0.001, *n* = 3. Scale bars = 20 µm

It has been reported that the Hippo/YAP signaling pathway prevents DNA damage-induced apoptosis through inhibition of the non-receptor tyrosine kinase C-Abl (Abelson murine leukemia viral oncogene) [49]. YAP activates pro-apoptotic genes along with p73 under conditions of DNA damage, and C-Abl promotes the association of YAP with p73, which induces apoptosis [50]. This program switching is mediated by C-Abl via phosphorylation of YAP at the Y357 residue [51, 52]. To investigate the relationship between C-Abl expression and neomycin-induced HC apoptosis in the mouse cochlea, we first verified the expression and localization of C-Abl by immunofluorescence staining, which showed that C-Abl was expressed in the nuclei of cochlear HCs (Fig. 6A). We next investigated the C-Abl expression in neomycin-treated cochleae and XMU/neomycin-treated cochleae. After 12 h of neomycin treatment, intense nuclear C-Abl staining was observed in neomycin-treated cochleae compared to the control cochleae, indicating active C-Abl signaling in response to neomycin exposure (Fig. 6B, C). Moreover, the immunofluorescence staining intensity of C-Abl was significantly decreased in XMU/neomycin-treated cochleae compared to neomycin-treated cochleae (Fig. 6B, C). qPCR and western blot results showed that C-Abl expression was significantly increased in neomycin-treated cochleae compared to control cochleae, and was significantly decreased in XMU/neomycin-treated cochleae compared to neomycin-treated cochleae (Fig. 7B–D). These results suggest that C-Abl expression is increased in cochlear HCs after neomycin exposure and that C-Abl expression in cochlear HCs is regulated by the Hippo/YAP signaling pathway.

### YAP overexpression inhibits C-Abl-mediated HC apoptosis in cochlear HCs after neomycin damage

Our initial findings suggested that C-Abl plays an important role in neomycin-induced HC apoptosis. To confirm the role of C-Abl in neomycin-induced HC apoptosis, we first dissected and cultured the cochleae from P3 WT mice and then treated them with neomycin together with XMU or VP (Fig. 7A). Immunofluorescence staining and western blot results demonstrated that C-Abl expression was significantly increased after neomycin treatment, and significantly decreased after pretreatment with XMU, indicating that YAP inhibits C-Abl activity (Figs. 6C, 7B). We also performed qPCR to explore the expression level of C-Abl signaling downstream genes in the cochlea after different treatments. The qPCR results demonstrated that the expression of the pro-apoptotic genes *C-Abl* and *p73* was significantly increased after neomycin treatment compared to the control group (Fig. 7C). Simultaneously, the expression of the pro-apoptotic genes *C-Abl* and *p73* was significantly increased after pretreatment of cochleae with the YAP inhibitor VP compared to the neomycin-only group. In contrast, the expression of the pro-apoptotic genes *C-Abl* and *p73* was significantly decreased when the cochleae were treated with the YAP agonist XMU (Fig. 7D). Thus, YAP/ p73-dependent apoptosis requires phosphorylation by C-Abl, which is in agreement with the findings of previous studies [53, 54]. In conclusion, neomycin-induced HC apoptosis was mediated by C-Abl, and YAP overexpression could inhibit C-Abl-mediated HC apoptosis in cochlear HCs after neomycin damage.

## Discussion

The Hippo signaling pathway is a highly evolutionarily conserved signaling pathway that was first discovered in Drosophila, and it is the most recently discovered member of the known signaling pathway families involved in the control of tissue development and organ size [21, 22]. The Hippo signaling pathway controls organ size by regulating cell proliferation, apoptosis, and stem cell self-renewal [55–57]. The core components of the Hippo signaling pathway include serine/threonine kinase cascades, transcriptional coactivators, and transcription factors. YAP is a key transcriptional coactivator downstream of the Hippo signaling pathway. Due to the activation of upstream kinases such as MST1/2 and LATS1/2, YAP phosphorylation occurs on the serine residues of five consistent HXRXXS motifs in vitro and in vivo [16, 58, 59]. YAP phosphorylation is located at the S127 site and interacts with 14-3-3 to inhibit the transcriptional activity of YAP, and this causes YA to remain in the cytoplasm and participate in the regulation of cell apoptosis. In contrast, when upstream kinases are inactivated, unphosphorylated YAP can bind to downstream TEAD family members (such as TEAD1-4) to enter the nucleus and participate in the regulation of cell proliferation, differentiation, and cell survival [60]. Protein phosphatase-1 specifically dephosphorylates YAP at S127 and promotes the nuclear accumulation and transcriptional activity of YAP [61]. Enhancing YAP expression contributes to wound repair and tissue regeneration after inflammatory injury [62].

Hearing loss is a common clinical sensory disorder affecting 466 million people worldwide and occurs mainly as a result of HC damage. Ototoxic drugs, viral infections, genetic susceptibility, aging, and noise can all lead to irreversible sensorineural hearing loss. Ototoxic drugs, especially aminoglycosides, cause permanent hearing loss in approximately half a million people each year in the USA [63]. Aminoglycosides enter HCs through specific membrane channels and endocytosis, and accumulate to cytotoxic levels, ultimately resulting in HC death [64, 65]. Because HCs do not have regenerative capacity in mature mammals, protection against HC damage is critical to maintaining hearing function. An increasing number of studies have focused on the mechanisms and therapeutic approaches of HC loss caused by ototoxic drugs [66], and the Hippo/YAP signaling pathway has been shown to play an important regulatory role in the repair and regeneration of tissues such as the eyes, brain, kidney, heart, liver, and skin [30–35]. Unfortunately, there are few studies on the role of the Hippo/YAP signaling pathway in auditory organs. Recently, YAP has been reported to be required for HC proliferation and differentiation in the inner ear [67–69]. However, whether the Hippo/YAP signaling pathway is required for HC survival has not been previously investigated.

To further explore the specific role and regulatory mechanism of the Hippo signaling pathway in HC damage protection in the mouse cochlea, we first verified the expression and localization of YAP in the mouse cochlea. We dissected cochleae from P3/P30 WT mice and cultured them in vitro. The immunofluorescence results showed that YAP was expressed in the cytoplasm of cochlear HCs, which is consistent with the biological characteristics of YAP, in which, under normal conditions, YAP remains in the cytoplasm and is targeted for degradation [70]. We next used the aminoglycoside drug neomycin to specifically damage HCs to construct an HC injury model. We found that the expression of YAP was decreased after neomycin injury. Hippo/YAP signaling pathway was activated by XMU in cochlear HCs after neomycin treatment, indicating that the Hippo/YAP signaling pathway is a protective physiological mechanism against neomycin injury.

We hypothesized that neomycin-induced HC loss could be prevented by regulating the expression level of YAP. To verify this hypothesis, we used the YAP agonist XMU and the YAP inhibitor VP to upregulate and downregulate the expression level of YAP, respectively. The pharmacokinetic characteristics of XMU could specifically inhibit the activity of MST1/2 in mice [71, 72], leading to activation of downstream YAP. VP has been used as a photosensitizer for photodynamic therapy, which has been approved by the Food and Drug Administration for treating age-related macular degeneration, pathological myopia, and neovascularization due to putative ocular histoplasmosis [73]. A recent high-throughput drug screening study reported that VP could inhibit the transcriptional activation of YAP-TEAD, leading to inhibition of cell proliferation and cell survival, and promotion of cell apoptosis [74].

In this study, we also found that the optimal concentration of XMU was 1 μM and that the optimal concentration of VP was 2 μM. The appropriate concentration of XMU or VP was used to pretreat the dissected cochleae, which were then cultured with neomycin in vitro. The immunofluorescence results showed that HC loss was significantly increased after neomycin injury compared to the control group. XMU significantly reduced the neomycin-induced HC loss, and VP significantly increased the neomycin-induced HC loss, indicating that neomycin-induced HC loss was significantly decreased after upregulation of YAP and increased after downregulation of YAP. These results are consistent with those of previous studies in other tissues such as the retina, brain, liver, kidney, and heart [30–35], all of which indicated that YAP plays a vital role in HC damage protection.

Several pathways are involved in aminoglycoside-induced HC damage, including cell apoptosis and oxidative stress [75, 76]. Previous studies have demonstrated that one of the most common causes of HC death is the production of ROS [77]. Oxidative stress inhibits the endogenous antioxidant systems of the cochlea, leading to excessive production of ROS, disrupting redox balance, triggering mitochondrial depolarization, and activating caspase-3-mediated apoptosis [78, 79]. In our study, we used MitoSox to detect the levels of ROS. Our results showed that XMU significantly reduced the ROS levels in cochlear HCs after neomycin exposure compared to the neomycin-only group, indicating that the Hippo/YAP signaling pathway can regulate the ROS levels. We next used TUNEL and Caspase-3 to label the apoptotic cells. The immunofluorescence results showed that the numbers of Myosin7a + /TUNEL + and Myosin7a + /Caspase3 + double-positive cells in the XMU/neomycin-treated group were significantly decreased compared to the neomycin-only group. In addition, the results of RT-qPCR demonstrated that the expression of the pro-apoptotic genes *caspase-8, Bax,* and *Apaf1* was significantly decreased, while the expression of the anti-apoptotic gene *Bcl-2* was significantly increased after XMU treatment. This suggests that the Hippo/YAP signaling pathway can regulate caspase-mediated apoptosis in cochlear HCs after neomycin injury. Activation of YAP inhibits HC apoptosis, while inhibition of YAP promotes HC apoptosis. In conclusion, the Hippo/YAP signaling pathway protects against neomycin-induced HC damage by reducing the ROS levels; thus, decreasing caspase-mediated cell apoptosis. However, this is not the sole mechanism responsible for aminoglycoside-induced HC death.

YAP acts as a tumor suppressor by activating p73 (a member of the downstream p53 family) in response to DNA damage. YAP binds to the PPPY motif of p73 through its WW domain and acts as a transcriptional coactivator of p73; thus, inducing the transcription of pro-apoptotic genes such as *Bax* [80]. C-Abl (Abelson murine leukemia viral oncogene) is a non-receptor tyrosine kinase that is activated to phosphorylate p73 at the Tyr99; thus, supporting the p73-dependent induction of apoptosis. At the same time, activated C-Abl also phosphorylates YAP at the Tyr357, and tyrosine-phosphorylated YAP accumulates on the targeted apoptotic gene promoter, where it preferentially binds to p73. Therefore, C-Abl-mediated phosphorylation of YAP increases the binding affinity of YAP to p73 by inhibiting the transcriptional activity of YAP-TEAD; thus, activating the pro-apoptotic target protein and transforming the activity of YAP from anti-apoptotic to pro-apoptotic [51, 52, 81]. To further investigate the effect of the C-Abl/ p73 transduction pathway in neomycin-induced HC damage in the mouse cochlea, we first verified the expression and localization of C-Abl. Immunofluorescence results showed that C-Abl was strongly expressed in the nucleus of HCs. In the following in vitro experiment, we observed the changes in major effector factors, such as C-Abl, YAP, TEAD, and p73, in different treatment groups at the RNA and protein levels. We found that the expression of C-Abl and p73 was significantly increased, while the expression of YAP and TEAD were decreased after neomycin injury. These findings indicate that neomycin exposure activates C-Abl and phosphorylates downstream YAP and that the phosphorylation of YAP leads to interaction with p73 and subsequent cell apoptosis and loss of HCs. Furthermore, YAP overexpression inhibited the activation of C-Abl; thus, inhibiting C-Abl-mediated cell apoptosis and suggesting that C-Abl-mediated HC apoptosis is regulated by the Hippo signaling pathway.

## Conclusion

In summary, we present the first investigation into the role of the Hippo/YAP signaling pathway in HC damage protection in the mouse cochlea. We showed that downregulated YAP expression increased neomycin-induced HC loss, and that YAP overexpression decreased neomycin-induced HC loss. We next demonstrated that the Hippo/YAP signaling pathway could regulate C-Abl-mediated HC apoptosis and ROS levels, which protects against neomycin-induced HC loss after neomycin exposure. Our results suggest that the Hippo/YAP signaling pathway plays an essential role in HC damage protection and thus represents a novel therapeutic target for aminoglycoside-induced HC injury.

## Data Availability

Not applicable.
